# Melatonin and Angelman Syndrome: Implications and Mathematical Model of Diurnal Secretion

**DOI:** 10.1155/2017/5853167

**Published:** 2017-12-12

**Authors:** Justyna Paprocka, Marek Kijonka, Piotr Wojcieszek, Marcin Pęcka, Ewa Emich-Widera, Maria Sokół

**Affiliations:** ^1^Department of Pediatric Neurology, School of Medicine in Katowice, Medical University of Silesia, Katowice, Poland; ^2^Department of Medical Physics, Maria Skłodowska-Curie Memorial Cancer Center and Institute of Oncology Gliwice Branch, Gliwice, Poland; ^3^Brachytherapy Department, Maria Skłodowska-Curie Memorial Cancer Center and Institute of Oncology Gliwice Branch, Gliwice, Poland; ^4^Faculty of Automatic Control, Electronics and Computer Science Biomedical Engineering, Silesian University of Technology, Gliwice, Poland

## Abstract

The main aim of the study was to compare the melatonin rhythms in subjects with Angelman syndrome (*n* = 9) and in children with (*n* = 80) and without (*n* = 40) epilepsy (nonepileptic patients diagnosed with peripheral nerve palsies, myopathy, and back pain) using our mathematical model of melatonin circadian secretion. The characteristics describing the diurnal hormone secretion such as minimum melatonin concentration, release amplitude, phase shift of melatonin release, and sleep duration as well as the dim light melatonin onset (DLMO) of melatonin secretion and the *γ* shape parameter allow analyzing the fit and deducing about how much the measured melatonin profile differs from a physiological bell-shaped secretion. The estimated sleep duration and phase shift of melatonin release as well as the DMLO offsets at 25% and 50% relative thresholds are the key characteristic of Angelman syndrome children. As revealed from the *γ* shape parameter, the melatonin secretion profiles are disturbed in majority of the AG subjects revealing rather a triangular course instead of the bell-like one.

## 1. Introduction

Angelman syndrome (AS) is a neurodegenerative disorder first described by English pediatrician Angelman in 1965. Population prevalence of Angelman syndrome has been calculated at 1 : 20,000–1 : 120,000 in children constituting 1.4% to 3% of all cases of severe mental retardation and about 6% of all cases with mental retardation with active epilepsy; both sexes are affected equally [1]. It is characterized clinically by a severe developmental delay, absence of speech, motor impairment, epilepsy, and a peculiar behavioral phenotype [2] as well as associated sleep disturbances and EEG abnormalities that can be used as an ancillary tool for Angelman syndrome patients [3]. The clinical diagnosis of the disease can be confirmed either by cytogenetic or DNA testing in about 80–85% of cases. In the vast majority of patients (65–75%), AS results from the loss of maternally imprinted chromosome region 15q11-13 or may be due to E6-P ubiquitin-protein ligase gene (*UBE3A*) mutation (5–11% patients), and in 2-3% of patients is caused by paternal uniparental disomy (*UPD*) [4–7]. In 1% of cases, a mutation in the imprinting center (*IC, ID*-imprinting defect) can be shown. It is worth noting that the chromosome region 15q11-q13 also contains genes for the *β*3, *α*5, and *γ*3 subunits of the *γ*-aminobutyric acid type A receptor (GABA_A_). GABAergic dysfunction (the shift in GABA_A_ receptor subunit expression and pharmacology) has been hypothesized to contribute to the occurrence of epilepsy and cognitive and behavioral impairments in this condition. Altered GABA_A_ receptor subunit expression may lead to more sensitive reaction to GABAergic agents used for insomnia treatment [8].

Recently the *γ*3 subunit gene has been suggested to link with autism [4–7]. In 2001, another gene—*ATP10C* (aminophospholipid-transporting ATP-ase gene)—was mapped to the AS “critical region,” distal to the *UBE3A* gene [4–7]. It is expressed in the brain and lymphoblasts, probably being involved in transferring phospholipids across the cell membrane.

There is imprecise and confusing data about melatonin diurnal profile in Angelman syndrome, although there is an agreement that sleep disturbances may be severe and appear frequently, in 90% of patients [9, 10]. That is why sleep problems are listed as “associated features” in the clinical diagnostic criteria for AS [11]. The observed sleep problems appear to be most profound between 2 and 6 years of age but may occur at any age [2], and the most frequently reported are the problems with sleep initiation (48%) and decreased need for sleep (42–49%) [8, 9, 12, 13]. Also, nocturnal seizures seen in AS may cause poor sleep maintenance and disrupt sleep architecture [9, 12, 14].

The management of sleep disorders in AS population is complex, and little high-quality data exist to guide a consistent approach to therapy. In insomniac patients with intellectual disabilities, melatonin supplementation is found to decrease sleep latency but does not influence sleep maintenance [15] In AS children, exogenous melatonin is claimed to decrease sleep latency and increase total sleep time as well [16]. When epileptic seizures are frequent —as in Angelman syndrome, Rett syndrome, and tuberous sclerosis—nocturnal seizures can cause poor sleep maintenance and the efficacy of melatonin treatment is lower. Moreover, its long-term treatment effects are still unknown.

In this paper, the focus is on the endogenous melatonin secretion patterns in children with Angelman syndrome. The characteristics describing the diurnal hormone secretion such as minimum melatonin concentration, release amplitude, phase shift of melatonin release and sleep duration as well as the dim light melatonin onset (DLMO) of melatonin secretion (being an important circadian marker) were estimated using our mathematical model of melatonin circadian secretion [17, 18]. The mathematical model has been added a new functionality—the parameter Γ(*t*_m_, *t*) [19], a modified Euclidean distance in time-concentration space—that allows to compare two sets of data in two-dimensional space. Gamma parameter is useful in fit analyzing and assessing the differences between the measured melatonin profile and a physiological bell shape. The AS model parameters were compared with those obtained for the children with and without epilepsy.

## 2. Materials and Methods

The study was approved by Medical University of Silesia's Ethics Committee. The informed written consents were taken from the parents or caregivers. The study was carried out at the Department of Pediatric Neurology, School of Medicine in Katowice, Medical University of Silesia in Katowice.

None of the studied subjects had taken any medications affecting melatonin secretion (benzodiazepines and their agonists, fluvoxamine, caffeine, vitamin B12, and nonsteroidal anti-inflammatory drugs: aspirin, ibuprofen, indomethacin, *α*-adrenolytics, prostaglandins inhibitors, calcium channel blockers, dexamethasone, and clonidine) before and during the study. Melatonin hypersecretion may be provoked by antidepressants like desipramine, fluvoxamine, and monoamine oxidase inhibitors and that is why the patients on such treatment were excluded from the study.

The patients with epileptic seizures on the day of sample collection were also excluded.

### 2.1. Angelman Syndrome Group (AG)

Nine subjects were included into the AG group. The inclusion criteria are based on clinical diagnosis and molecular examinations. In all children, the genetic study confirmed 15q11.2 deletion. The patients' age at the time of diagnosis ranged from 3 to 10 years (mean 5.55 years, female to male ratio was 4 : 5). The follow-up period varied from 2 to 4 years (mean 2.5 yrs).

Table 1 shows the medical characteristics of the children. Epilepsy was diagnosed in all AS patients. The main types of seizures were myoclonic and generalized tonic-clonic and absence seizures. In all AS children, EEG showed generalized paroxysmal activity (with temporal and parietal predominance) and abnormal sleep pattern. Among antiepileptic drugs used in the Angelman patients with epilepsy were valproic acid and lamotrigine. Brain MRI was normal in 2 children, whereas in the remaining children the brain abnormalities were seen (Table 1).

All AG patients had circadian rhythm sleep disorders (CRSD): irregular sleep-wake type, *n* = 3; free-running type, *n* = 2; and delayed sleep phase type, *n* = 4.

#### 2.1.1. Epilepsy Group

The epilepsy group included 80 patients at the mean age of 5 years 6 months; female to male ratio was 42 : 38. Patients with epilepsy were reviewed for the following seizure type and syndrome: seizure frequency, age at seizure onset, electroencephalogram tracings, current and previous AEDs, seizure timing, etiology, cognitive status, and family history. The type of epileptic seizures was defined following the International League Against Epilepsy Classification and Terminology. The mean duration of epilepsy was about 4.7 years (range 2 months–17 years). Antiepileptic drugs used were as follows: valproic acid (*n* = 64, 82%), lamotrigine (*n* = 22, 28.2%), and levetiracetam (*n* = 20, 25.6%).

#### 2.1.2. Comparison Group (CG)

The comparison group (CG) was constituted of 40 nonepileptic patients (mean age was 6 years and 11 months) female to male ratio was 24 : 16. Among the patients, peripheral nerve palsy (facial nerve palsy *n* = 18, 45%), myopathy (*n* = 11, 27.5%), and back pain (*n* = 11, 27.5%) were diagnosed.

### 2.2. Statistical Analysis

The AG, EG, and CG groups were homogeneous as regards age (*p* = 0.9256, Kruskal–Wallis test) and intellectual development (*p* = 0.4801, Kruskal–Wallis test).

Since the individual group sizes differ markedly and also the groups do not meet the requirements for parametric tests (the data is not normally distributed), in order to compare the AG model parameters with those obtained for the EG and CG groups, a nonparametric Mann–Whitney *U* test and Wilcoxon test was used. The *p* values less than 0.05—a predetermined significance level—were accepted as indicating that the observed result would be highly unlikely under the null hypothesis. To explore the intragroup variability of the AG group, qualitative research was also applied.

### 2.3. Blood Sampling

In order to enable the comparison of the AG melatonin secretion patterns with those obtained previously for EG and CG [17, 18], we decided to use blood as a material for the analyses.

The blood samples were drawn every 3 hours through an intravenous catheter. During night hours, the blood samples were taken by red dim light. The melatonin level was obtained using radioimmunoassay (RIA) method.

### 2.4. MLT Secretion Model

The individual parameters of the melatonin diurnal cycle were obtained using our mathematical model developed for children with epilepsy [17] and tuberous sclerosis complex [18]. In this model, the time dependency of the melatonin concentration can be described by the MLT(*t*) function:

(1) $$\begin{array}{c} MLT\left(t\right)=b_{1}+b_{2}\exp\left(-\frac{{\left(\cos\left(\left(\pi /12\right)t-\left(\pi /12\right)b3\right)-1\right)}^{2}}{{\left(\cos\left(\left(\pi /24\right)b_{4}\right)-1/\sqrt{\ln2}\right)}^{2}}\right), \end{array}$$

where *b*_1_ denotes a minimum melatonin concentration (pg/mL), *b*_2_ is a melatonin release amplitude (pg/mL), *b*_3_ is a phase shift of melatonin release (h), and *b*_4_ is sleep duration (represented by the full width at half maximum (FWHM) of melatonin secretion model) (h). Maximum melatonin concentration (*b*_max_) is given by the sum of *b*_1_ and *b*_2_. The *b*_3_ and *b*_4_ parameters enable DLMO characteristics to be estimated [18]: DLMO onset at the 50% relative threshold, DLMOon_50_ = *b*_3_ − (*b*_4_/2), DLMO offset at the 50% relative threshold, DLMOoff_50_ = *b*_3_ + (*b*_4_/2), DLMO onset at the 25% relative threshold, DLMOon_25_ ≈ *b*_3_ − (2/*π*)*b*_4_, and DLMO offset at the 25% relative threshold, DLMOoff_25_ ≈ *b*_3_ − (2/*π*)*b*_4_.

In the current work, additional functionality was added to our modelling tool to allow comparison of the data distribution and to obtain information on the degree of disturbance from the bell-shaped secretion pattern (such as a triangular secretion course and the diurnal fluctuations of melatonin concentration). Shape fitting accuracy was defined as the maximum normalized Euclidean distance of the measured values from the bell course secretion. The circadian rhythm function MLT(*t*) was differentiated in each measurement point MLT_m_(*t*_m_). The normalized Euclidean distance from the bell model in time point *t*_m_ was calculated as

(2) $$\begin{array}{c} \gamma \left(t_{m}\right)=\min\left\{\Gamma \left(t_{m},t\right)\right\}\forall t\in \left[0,24\right), \end{array}$$

where Γ(*t*_m_, *t*) is the modified Euclidean distance of the measurement point MLT_m_(*t*_m_) from the model MLT(*t*) in time-concentration space [19]:

(3) $$\begin{array}{c} \Gamma \left(t_{m},t\right)=\sqrt{\frac{{\left(t_{m}-t\right)}^{2}}{\delta {t_{m}}^{2}}+\frac{{\left({MLT}_{m}\left(t_{m}\right)-MLT\left(t\right)\right)}^{2}}{\delta {{MLT}_{m}}^{2}}}. \end{array}$$

The parameters *δt*_m_ and *δ*MLT_m_ are the standardization factors determined so that the shape accuracy factor *γ*(*t*_m_) is equal to 1 when the distance of the measurement point MLT_m_(*t*_m_) from the model function MLT(*t*) is equal to *δt*_m_ or *δ*MLT_m_. Their values were taken as 20 min and 5% of the measurement value in point *t*_m_, respectively. In other words, if the distance of the measurement point MLT_m_(*t*_m_) from the bell model MLT(*t*) exceeds certain values, the function *γ*(*t*_m_) is greater than 1.

Then, the highest value of the shape mismatch parameter was used for the analysis:

(4) $$\begin{array}{c} \max\gamma =\max\left\{\gamma \left(t_{m}\right)\right\}\forall \left\{t_{m}\right\}. \end{array}$$

Its value should not exceed 2 in case of a physiological bell melatonin secretion, and for an ideal course with a clearly marked amplitude phase and with a well-marked daytime plateau, a value below 1 is expected. Any secretion disturbances, manifesting in the form of fluctuations and/or peaks, in triangular course result in the max*γ* increase above 2.

The obtained model parameters *b*_1_, *b*_2_, *b*_3_, *b*_4_, *b*_max_, DLMOon_50_, DLMOoff_50_, DLMOon_25_, DLMOoff_25_, and max*γ* were compared for the studied groups and subjected to statistical analysis.

## 3. Results

The quality of the obtained models was verified by the normality test of the residuals' distribution, statistical significance of the estimated parameters, percentage of the explained variance (>81%), and the *R* value (>0.90).

**Figure 1:**
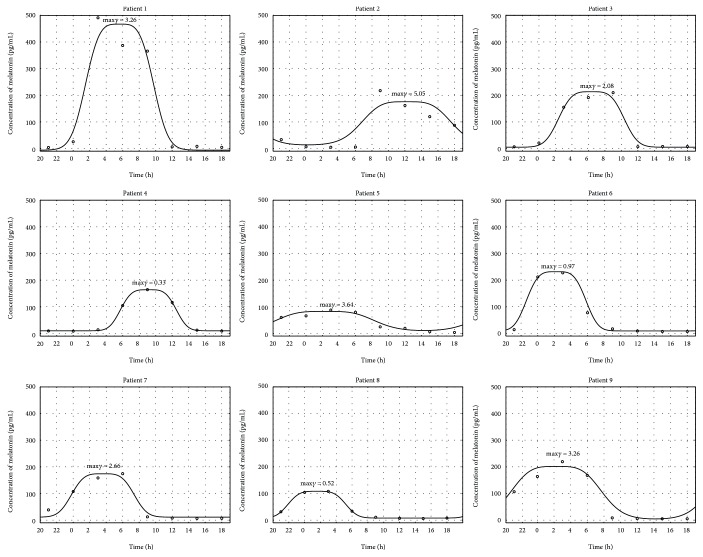
Melatonin secretion for 9 patients from the AG group: solid line—estimated model, circles—measured values. The obtained maximum gamma values are given above the models.

The melatonin secretion models calculated for 9 patients from the AG group based on the measured melatonin concentration values are highly variable with respect to the characteristics of the model MLT(*t*) curves. For 67% patients of the AG group (the patients 1, 2, 3, 5, 7, and 9), max*γ*—the values of this parameter are shown in Figure 1 above the modeled function—is higher than 2, which means that melatonin secretion is severely disturbed in the AG group.

**Figure 2:**
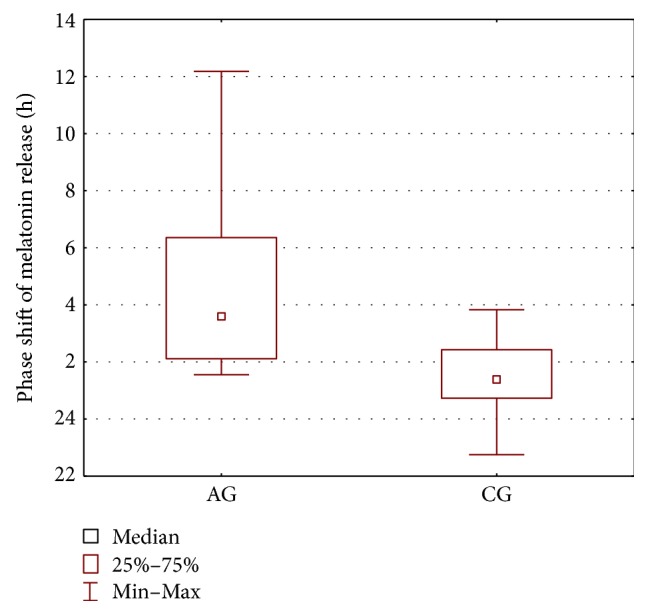
Boxplot of the phase shift of melatonin release (represented by the *b*_3_ parameters) for the AG and CG groups.

**Figure 3:**
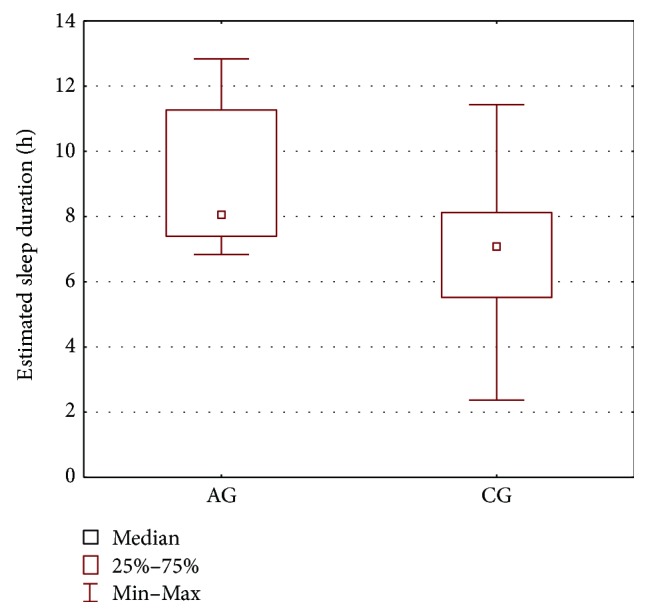
Boxplot of the estimated sleep duration (represented by the *b*_4_ parameters) for the AG and CG groups.

**Figure 4:**
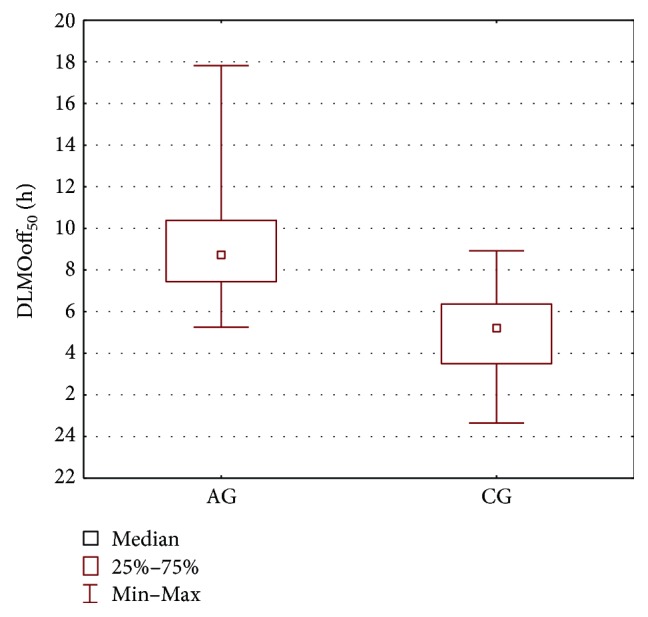
Boxplot of the DLMOoff_50_ parameter for the AG and CG groups.

**Figure 5:**
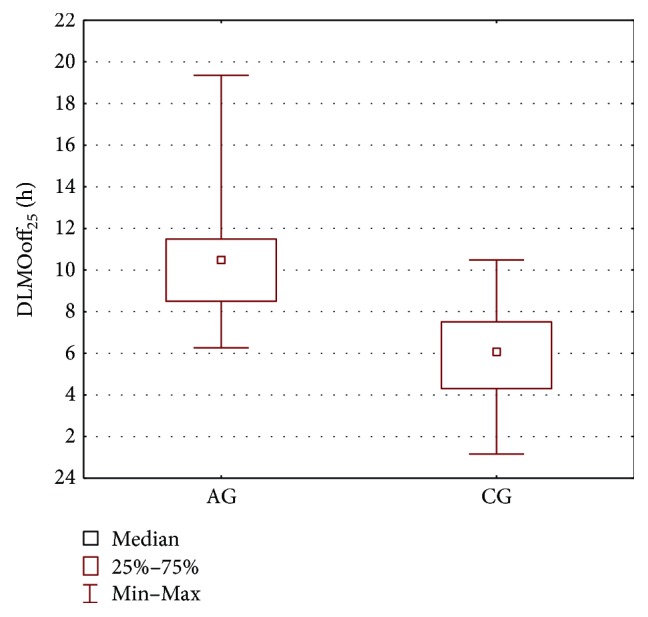
Boxplot of the DLMOoff_25_ parameter for the AG and CG groups.

In order to compare the AG group and the CG group, the parameters' estimates obtained for the melatonin secretion models were subjected to statistical analysis. The Mann–Whitney *U* test was applied and the results are presented in Table 2 and Figures 2, 3, 4, and 5. As seen from the comparison, the difference in phase shifts (Figure 2) is significant (*p* < 0.005) and sleep duration (Figure 3) is longer in Angelman syndrome (*p* < 0.05). The statistical tests indicate also that DLMOoff_50_ (Figure 4) and DLMOoff_25_ (Figure 5) differ significantly (*p* < 0.005) for both groups. Interestingly, the max*γ* parameter does not differentiate the AG and CG groups.

**Figure 6:**
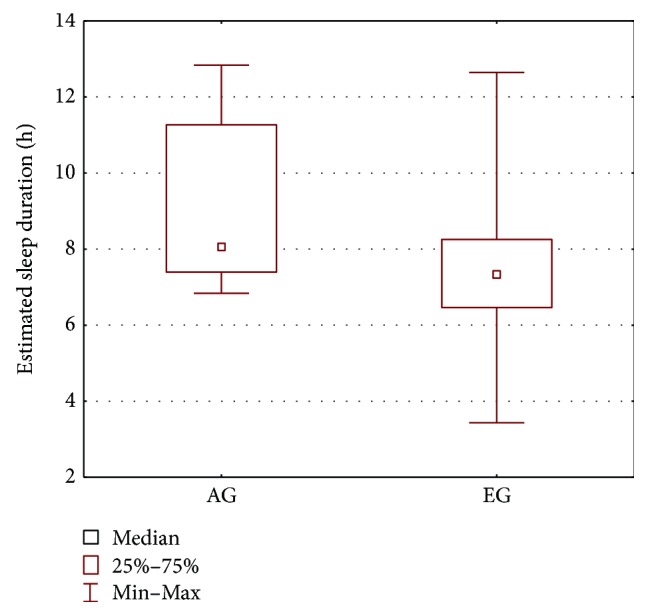
Boxplot of the estimated duration of sleep for the AG and EG groups.

**Figure 7:**
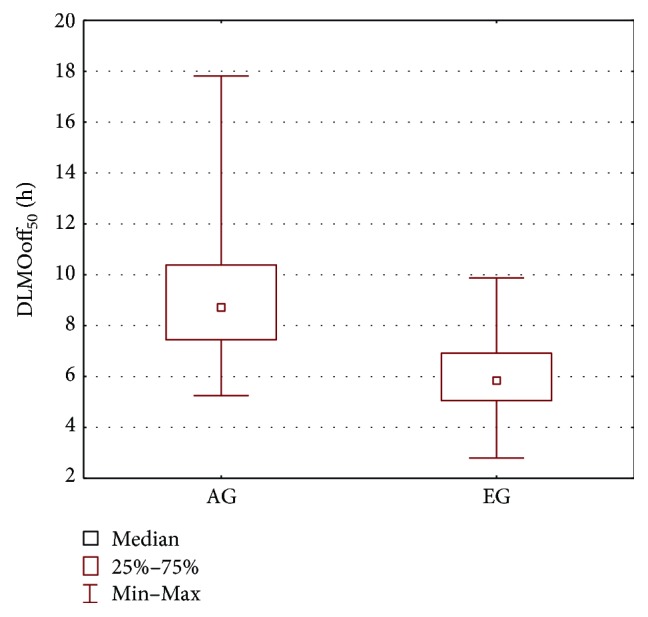
Boxplot of the DLMOoff_50_ parameter for the AG and EG groups.

**Figure 8:**
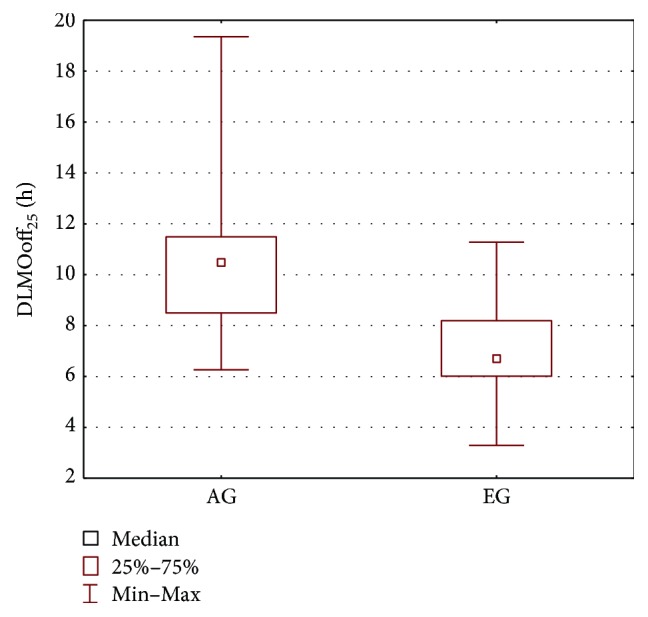
Boxplot of the DLMOoff_25_ parameter for the AG and EG groups.

Since all patients of the AG group present with epileptic seizures, the AG group was also compared with the epileptic EG group. The results of the Wilcoxon test and Mann–Whitney *U* test are shown in Table 3 and in Figures 6, 7, and 8. The parameters that differentiate the AG and EG groups are *b*_4_—the estimated sleep duration (Figure 6), DLMOoff_50_ (Figure 7), and DLMOoff_25_ (Figure 8). Similarly, as in the AG versus CG comparison, the max*γ* parameter does not differentiate the AG and EG groups, but the *p* value is much lower (*p* = 0.078 for AG versus EG, whereas *p* = 0.3906 for AG versus CG).

Thus, the comparison of the AG and EG groups indicates the same parameters as for AG versus CG to be significant—the only difference is that the *p* value for the phase shift of melatonin release comparison—being 0.0521—only slightly missed the margin of significance.

When compared to the EG and CG groups, the Angelman syndrome patients exhibit elongated sleep duration and a strong shift of the dim light melatonin offsets, DLMOoff_25_ and DLMOoff_50_, to later hours. Thus, the offset markers of the timing of the circadian clock are rather important, not the onset ones.

## 4. Discussion

The AG group was homogeneous with respect to the type of mutation and with respect to antiepileptic drugs used. According to the genotype-phenotype correlation by Lossie et al. [20], all children with a deletion within chromosome 15 are in class I, which includes severely affected patients with seizures, microcephaly, hypopigmentation, and severe developmental delay.

The main aim of the study was to compare the melatonin rhythms in subjects with Angelman syndrome and—because all children in our AG group present with epilepsy—in children with and without epilepsy (nonepileptic patients diagnosed with peripheral nerve palsies, myopathy, and back pain). To the best of our knowledge, this is the first study that explores the AS diurnal melatonin secretion patterns using mathematical modeling. The model of melatonin secretion provides a set of parameters directly characterizing melatonin cycle, such as minimum melatonin concentration, melatonin release amplitude, phase shift of melatonin release, estimated sleep duration, and DLMO onsets and offsets, which could be of potential clinical usefulness as factors facilitating classification of sleep disturbances. Such approach enables the individual and group average secretion patterns to be analyzed in an objectified way, by comparing various model parameters, including DMLOs. This is in accord with the need for consistent standards and more rigorous study of sleep in individuals with AS emphasized in the recent review study [21]. Our results show that the estimated sleep duration and phase shift of melatonin release as well as the DMLO offsets at 25% and 50% relative thresholds are the key characteristic of Angelman syndrome children.

Children with Angelman syndrome may present with sleep onset insomnia as well as sleep maintenance problems, and low endogenous melatonin levels are often claimed to be an essential feature of melatonin secretion in their circadian rhythms [22–24].

This key observation—low melatonin—has not been satisfactorily explained yet. It has been hypothesized, but not confirmed, that this might be due to decreased production of melatonin, decreased expression of its receptors, or other factors determining sensitivity to this hormone in Angelman syndrome [23]. Another explanation of low melatonin involved the use of valproate [16], as this medication is known to suppress plasma melatonin levels [25]. However, such supposition was rejected by Braam et al. [23], who compared the endogenous melatonin levels in children administered with valproate and those who did not use it, and by Takaesu et al. [24], who observed minimal impact of sodium valproate on the low serum levels. On the other hand, it cannot be excluded that the effects of valproate treatment may differ in AS patients and controls. Unfortunately, the sample sizes in all AS studies are small and often age-inhomogeneous; thus, the melatonin levels may have been confounded by including adult subjects into the studied children groups (like in [24]), and the effects of such mixing may overshadow the variability and reduce the statistical power to detect differences. The latter factor—age-homogeneity—seems to be especially of importance for children groups. When analyzing the melatonin secretion rhythms in children, it should be taken into account that melatonin concentration declines during childhood—melatonin production commences, becomes circadian, and reaches its highest nocturnal blood levels between the ages of one to three years. During the remainder of childhood, nocturnal peak levels drop progressively by 80%. Such age-related behavior was observed for healthy children [26] as a statistically significant linear decreasing trend in peak melatonin from 175 ± 109 pg/mL to 128 ± 44 pg/mL during the course of puberty in children from 5 to 17 years of age. One of the explanations attributed this effect to the alterations in body size during development [27]. Interestingly, in our study of the age-homogeneous AG group (age range 3–10 years), the median value of maximum melatonin concentration (*b*_max_, given by the sum of *b*_1_ and *b*_2_) is 175.69 pg/mL and falls within the range reported by Cavallo for the younger children. Thus, the maximum concentration of melatonin in our group is not lower than that for the healthy children, being even higher than the *b*_1_ + *b*_2_ values for EG (117.38 pg/mL) and CG (152.14 pg/mL).

The melatonin secretion AS profiles are, however, evidently disturbed. Instead of the bell-shaped secretion, expected for physiological conditions, a triangular course is dominant in the AG group—the *γ* parameter exceeds 2 for 67% of the subjects. Since there is no statistical difference of the max gamma values for the AG versus EG and AG versus CG comparisons, the shape disturbances are also expected in case of the epileptic and nonepileptic groups.

Although the MLT(*t*) function shape disturbance is a common feature for the studied groups, the AG model characteristics differ from the CG and EG ones with respect to phase shift of melatonin release (*b*_3_) and estimated sleep duration (*b*_4_), but the DLMO offset parameters (because they are expressed by the sum of *b*_3_ and *b*_4_) are the strongest differentiating parameters. The dim light melatonin onset (DLMOon), the point in time when melatonin levels begin to rise in the evening, and the dim light melatonin offset (DLMOoff), the point in time when melatonin levels diminish in the morning, are two commonly derived circadian phase markers [28]. DLMOon is claimed to be the most reliable circadian phase marker, more reliable than the DLMOoff and phase markers derived from the core body temperature rhythm [29]; however, in case of our study, the melatonin offset revealed to be of statistical importance distinguishing Angelman syndrome patients from the other two, EG and CG, groups. It seems that occurrence of two simultaneous features of melatonin secretion disorder in the studied Angelman group—elongated sleep duration and a strong phase shift of the melatonin amplitude—are responsible for a compensation of the secretion DLMOon parameters to the values similar as for CG and for the displacement of the DLMOoff parameters. Moreover, we decided to use the relative thresholds in our modelling calculations, since they allow normalizing the amplitude differences in analysis. Thus, such approach facilitates the comparisons between the individuals of strongly varying amplitudes (of maximum melatonin levels) [30].

The DLMO parameters obtained for the AG group differ than those for the healthy children. Normally, melatonin rises between 7 pm and 9 pm in children 6 to 12 years of age [31], peaks between 2 am and 4 am, then gradually decline [32]. Delay in circadian phase, in addition to delayed sleep-wake times (sleep duration problem) are characteristic for delayed sleep-wake phase disorder, the circadian rhythm disorder frequent in Angelman syndrome [21, 24, 33].

In Angelman syndrome, epilepsy is present in 80–90% of patients (in our AG group, all children were diagnosed with epilepsy). Though, it is expected that circadian clock plays an important role in epileptic patients, however, still relatively little is known. In humans, the effect of epilepsy on melatonin and vice versa has been described in several studies; however, the results are conflicting. Some authors describe low-baseline levels of melatonin in people with epilepsy [34, 35], whilst others report elevated levels with a maintained day-night rhythm [36] or with a phase difference similar as in controls [37]. On the other hand, normal plasma melatonin curve in epilepsy patients under dim lit conditions [38] as well as in the study involving epileptic children [39] was found. Our results obtained for children with epilepsy (the EG group) show that the maximum melatonin concentration (*b*_max_, given by a sum of *b*_1_ and *b*_2_) is the lowest (117.38 pg/mL) among the studied groups, and the phase shift in melatonin release occurs later as compared to the CG group, but earlier than for AG. In patients with epilepsy, melatonin concentration is reported to be slightly increased or unchanged in comparison to healthy subjects [34, 35, 38], but antiepileptic treatment itself may influence melatonin secretion [40].

The comparison of the AG and CG groups seems to resemble the observations of Wirrell et al. obtained for epileptic [38] and nonepileptic siblings [41]. In children with epilepsy and mental retardation, these authors report significantly greater sleep problems than for their nonepileptic siblings with normal cognitive function. However, the insignificance of the max*γ* parameter indicates that the shape disturbances of the melatonin secretion are also frequent in the CG group.

The confirmation of sleep disturbances in people with AS—as being significantly related to the presence of seizures—comes also from other studies [13, 25, 42, 43].

One of the limitations of our study is unknown drug influence on the melatonin cycle. Valproic acid may lower melatonin secretion in epileptic patients. On the other hand, some antiepileptic drugs like lamotrigine and levetiracetam may have a positive effect on the sleep structure resulting in more REM and slow-wave sleep [44]. The direct effect of AEDs on sleep is difficult to measure because of many confounding factors, with leading one-polypharmacy [44–46].

Another limitation of this study is small sample size in case of the AG group. Therefore, the application of the statistical analyses may be limited. It is, however, extremely difficult to include a large number of participants with Angelman syndrome in any randomized controlled trial, because Angelman syndrome is a relatively rare genetic disorder.

## 5. Conclusions

Mathematical modeling of circadian melatonin cycle facilitates statistical analysis of the patients' hormone levels offering a set of parameters that enable objectification of the secretion description. The comparison of the melatonin secretion data and the mathematical model parameters revealed statistically significant differences between the children with Angelman syndrome and the children with epilepsy and the nonepileptic ones (with peripheral nerve palsies, peroneal nerve palsy, myopathy, and back pain).

The estimated sleep duration and phase shift of melatonin release as well as the DMLO offsets at 25% and 50% relative thresholds are the key characteristic of Angelman syndrome children.

As revealed from the *γ* shape parameter, the measured melatonin secretion profiles are disturbed in the majority of the AS subjects from the AG group revealing a triangular course instead of a bell-like one.

These results confirm that a variety of sleep problems may exist in a significant portion of individuals with Angelman syndrome, most prominently in the areas of sleep-wake patterns and sleep duration.

## Figures and Tables

**Table 1:** Children with Angelman syndrome. The characteristics.

Age at diagnosis (sex)	Patient 1 10 yrs (f)	Patient 2 4 yrs (f)	Patient 3 3 yrs (f)	Patient 4 5 yrs (m)	Patient 5 5 yrs (f)	Patient 6 4 yrs (m)	Patient 7 3 yrs (m)	Patient 8 7 yrs (m)	Patient 9 9 yrs (m)
Seizure type	TS, AA	IS, MS, AT, AA	MS, AA	GTCS, AA, AT	AA, GTCS	MS, AA, GTCS	MS, AA	MS, AA, GTCS	MS, AA, GTCS
EEG	Abnormal sleep pattern, generalized abnormalities								
	With the left side predominance	Hipsarrhythmia	With the frontal and temporal predominance	With the temporal and occipital predominance	With the temporal and occipital predominance	With the frontal and temporal predominance	With the right side predominance	With the left side predominance	With the frontal and temporal predominance
AEDs	VPA, LTG	VPA, LTG	VPA, LTG	VPA, LTG	VPA, LTG	VPA, LTG	VPA, LTG	VPA, LTG	VPA, LTG
Neurological examination	Microcephaly, gait ataxia	Microcephaly, spastic paraparesis, ataxia	Microcephaly, ataxia	Microcephaly, spastic quadriparesis	Microcephaly, spastic paraparesis, ataxia	Microcephaly, spastic paraparesis	Microcephaly, ataxia	Microcephaly, ataxia, spastic quadriparesis	Microcephaly, spastic quadriparesis
DQ/IQ (according to revised Brunet-Lezine or Wechsler scale)	72	27	49	17	32	65	52	20	24
Brain MRI	Normal	Delayed myelination	Normal	Delayed myelination, widening of the perivascular spaces of Virchow-Robin	Normal	Delayed myelination	Normal	Delayed myelination	Frontal and temporal cortical atrophy, narrow corpus callosum, and widening and asymmetry of the ventricular system
Minimum melatonin concentration (pg/mL)	0.1787	8.2274	4.8639	10.0281	2.53770	9.2324	11.2680	7.8354	0.3444
Melatonin release amplitude (pg/mL)	466.7658	167.4662	208.3878	153.9621	79.88783	222.4951	162.7621	100.3606	201.1275
Phase shift of melatonin release (h)^∗^	5.6183	12.1787	6.3585	9.1097	2.30309	2.1005	3.5904	1.5535	2.1150
Estimated sleep duration (h)^∗^	8.402	11.2672	8.0544	6.8388	12.83515	7.2964	7.7087	7.3945	11.4236
Maximum melatonin concentration (pg/mL)	466.9445	175.6936	213.2517	163.9902	82.4255	231.7275	174.0301	108.1960	201.4719
DLMOon_50_ (h)^∗^	1.4173	6.5451	2.3313	5.6903	19.8855	22.4523	23.7360	21.8562	20.4033
DLMOoff_50_ (h)^∗^	9.8193	17.8123	10.3857	12.5291	8.7207	5.7487	7.4447	5.2507	7.8268
DLMOon_25_ (h)^∗^	0.2667	5.0021	1.2283	4.7537	18.1278	21.4531	22.6803	20.8436	18.8389
DLMOoff_25_ (h)^∗^	10.9699	19.3553	11.4887	13.4656	10.4783	6.7479	8.5004	6.2633	9.3912
Max gamma	3.2600	5.0490	2.0780	0.3255	3.6410	0.9718	2.6570	0.5221	3.2590

TS: tonic spasms; MS: myoclonic seizures; GTCS: generalized tonic-clonic seizures; AA: atypical absences; AT: astatic seizures; IS: infantile seizures; VPA: valproic acid; WGB: vigabatrin; LTG: lamotrygine; TPM: topiramate. ^∗^Time in decimal scale.

**Table 2:** Results of Mann–Whitney *U* test for the AG and CG groups (the statistically important values are in bold).

Model parameters	Median of the CG group	Median of the AG group	*p* value
*b*_1_, minimum melatonin concentration (pg/mL)	6.16	7.83	0.6065
*b*_2_, melatonin release amplitude (pg/mL)	142.51	167.46	0.372
*b*_3_, phase shift of melatonin release (h)^∗^	1.38	3.59	**0.0039**
*b*_4_, estimated sleep duration (h)^∗^	7.07	8.05	**0.0427**
Maximum melatonin concentration (pg/mL)	152.14	175.69	0.372
DLMOon_50_ (h)^∗^	22.1	23.74	0.1592
DLMOoff_50_ (h)^∗^	5.2	8.72	**0.0005**
DLMOon_25_ (h)^∗^	21.33	22.69	0.2039
DLMOoff_25_ (h)^∗^	6.06	10.47	**0.0004**
Max gamma	1.81	2.65	0.3906

^∗^Time in decimal scale.

**Table 3:** Results of Mann–Whitney *U* test for the AG and EG groups (the statistically important values are in bold).

Model parameters	Median of the EG group	Median of the AG group	*p* value
*b*_1_, minimum melatonin concentration (pg/mL)	5.84	7.83	0.7675
*b*_2_, melatonin release amplitude (pg/mL)	116.34	167.46	0.064
*b*_3_, phase shift of melatonin release (h)^∗^	2.34	3.59	0.0521
*b*_4_, estimated sleep duration (h)^∗^	7.32	8.05	**0.0421**
Maximum melatonin concentration (pg/mL)	117.38	175.69	0.0673
DLMOon_50_ (h)^∗^	23.02	23.74	0.298
DLMOoff_50_ (h)^∗^	5.83	8.72	**0.0016**
DLMOon_25_ (h)^∗^	22	22.69	0.3507
DLMOoff_25_ (h)^∗^	6.69	10.47	**0.001**
Max gamma	1.02	2.65	0.078

^∗^Time in decimal scale.
